# Characteristics of Gastrointestinal Bleeding While Taking Direct Oral Anticoagulants in Patients with Nonvalvular Atrial Fibrillation and Differences Among Drugs—A Single-Center Retrospective Cohort Study

**DOI:** 10.3390/jcm14010095

**Published:** 2024-12-27

**Authors:** Naoaki Aoki, Koichiro Abe, Haruka Tokutomi, Kohei Kajita, Masayuki Sone, Taku Honda, Hitoshi Aoyagi, Akari Isono, Kumiko Konno, Ken Kozuma, Toshihiko Arizumi, Yoshinari Asaoka, Shinya Kodashima, Takatsugu Yamamoto, Atsushi Tanaka

**Affiliations:** Department of Medicine, Teikyo University School of Medicine, Tokyo 173-8605, Japan; naoakiaoki0527@med.teikyo-u.ac.jp (N.A.);

**Keywords:** anticoagulants, gastrointestinal bleeding, lower gastrointestinal tract, peptic ulcer, atrial fibrillation

## Abstract

**Introduction**: Direct oral anticoagulants (DOACs) are frequently used to prevent embolism in atrial fibrillation. Gastrointestinal bleeding is frequent, but its drug-specific characteristics remain unclear. This study examined the frequency and characteristics of gastrointestinal bleeding in patients with nonvalvular atrial fibrillation for different DOACs. **Methods**: The present study included 978 patients receiving treatment with DOACs for nonvalvular atrial fibrillation between 2011 and 2018 and examined the frequencies of clinically significant events including major cardiovascular and cerebrovascular events, hemorrhagic events, or death during the first 5 years of prescription. Gastrointestinal bleeding was evaluated for the frequency, source and severity of gastrointestinal bleeding by DOAC type. **Results**: The median age of subjects was 73 years (interquartile range, 65–80 years), and 622 (64%) were male. The overall observation period was 2499 person-years. During this period, 102 (4.1/100 person-years) major cardiovascular events and 107 (4.3/100 person-years) clinically significant bleeding were reported, including 60 cases (2.4/100 person-years) of gastrointestinal bleeding and 37 cases (1.5/100 person-years) of all-cause mortality. Gastrointestinal bleeding accounted for more than half of all bleeding events among DOAC users, and bleeding from the lower gastrointestinal tract was more common than that from the upper gastrointestinal tract, particularly with dabigatran. No significant difference was seen in the rate of gastrointestinal bleeding by drug type. Peptic ulcer accounted for half of the events of upper gastrointestinal bleeding, all of which were severe. **Conclusions**: Gastrointestinal bleeding is frequent among patients taking DOACs, especially from the lower alimentary tract. The tendency was more pronounced with dabigatran. In the upper gastrointestinal tract, severe peptic ulcer bleeding is common and requires caution.

## 1. Introduction

The number of patients with atrial fibrillation and embolic complications is increasing with the aging of society [1,2]. Direct oral anticoagulants (DOACs) are frequently used for the prevention of embolism, and they have been reported to be as effective as or more effective than conventional warfarin in preventing embolism [3,4,5,6]. Particularly in Asian populations, DOACs have been reported to have a lower risk of bleeding and a higher safety profile than warfarin [7,8,9]. On the other hand, gastrointestinal bleeding during DOAC administration has been reported to be as frequent or more frequent than with warfarin, posing a major clinical challenge [10,11,12,13,14]. We have previously reported that gastrointestinal bleeding is the most frequent bleeding event among DOAC users [15], that the rate of gastrointestinal bleeding is higher in the very elderly [16], that the discontinuation of DOACs after bleeding increases the incidence of serious embolism [17], and that polypharmacy carries a significant risk of gastrointestinal bleeding [18]. However, most previous reports, including our study, have focused on the frequency of gastrointestinal bleeding and overall risk factors, and there is insufficient information on the details of gastrointestinal bleeding in people taking DOACs. Given the importance of gastrointestinal bleeding in DOAC treatment, such information would contribute greatly to the management of DOAC users in clinical practice.

DOACs have been suggested to show different pharmacological characteristics depending on the drug, and differences in clinical efficacy are likely to exist [19,20]. However, clinical data on differences in the frequency of gastrointestinal bleeding have not yielded clear conclusions with some reporting differences [21,22,23,24,25,26,27,28,29,30,31] and others reporting none [32,33,34]. In addition, few reports have described the frequency of bleeding among patients with diseases causing bleeding. In addition, little information is available on the causes of bleeding in both cases, and the details are unclear [15,35]. This study investigated the incidence of bleeding events and embolisms, including gastrointestinal bleeding, among patients taking DOACs in clinical practice, and it also examined the characteristics of gastrointestinal bleeding according to the drug taken.

## 2. Methods

**Figure 1:**
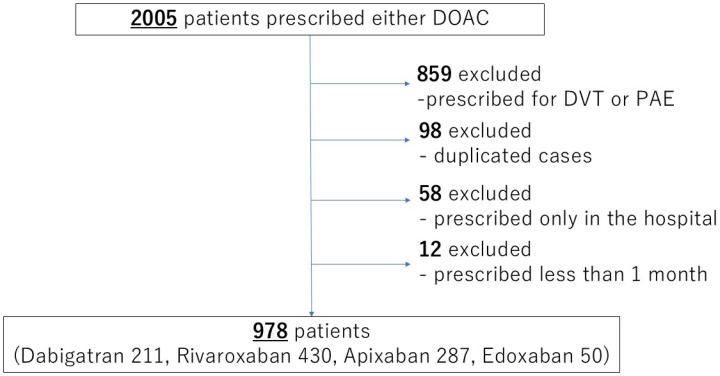
Flowchart for selection of study subjects.

This was a single-center, retrospective study of subjects selected from a list of patients at Teikyo University Hospital. The purpose of this study was to evaluate the incidence of adverse events in patients with nonvalvular atrial fibrillation who were treated with DOACs in a stable state, and the subjects were selected in the following manner. First, from April 2011 to June 2018, all patients who had been prescribed any of the DOACs (Dabigatran etexilate; Prazaxa (Nippon Boehringer Ingelheim Co., Ltd., Tokyo, Japan), Rivaroxaban; Xarelto (Bayer Yakuhin, Ltd., Osaka, Japan), Apixaban; Eliquis (Bristol Myers Squibb, Tokyo, Japan), or Edoxaban; Lixiana (Daiich-Sankyo Inc., Tokyo, Japan) were selected from among all patients who had received prescriptions for nonvalvular atrial fibrillation. Cases with prescriptions for issues other than nonvalvular atrial fibrillation, prescriptions only for hospitalization, or prescriptions for less than 1 month were excluded from the study. In cases with multiple DOAC prescriptions, only the period corresponding to the prescription of the first drug was included in the observation. In the end, 978 patients were included in the study (Figure 1). For each subject, we investigated clinically significant bleeding complications (bleeding academic research consortium criteria type 2 or higher) [36], gastrointestinal bleeding, major adverse cardiovascular and cerebrovascular events (MACCEs) of cardiac death, vascular death, nonfatal myocardial infarction, nonfatal stroke, or major artery occlusion, and death during the first 5 years of prescription. The onset of gastrointestinal bleeding was defined as bleeding of obvious gastrointestinal origin, such as hematemesis, coffee-ground vomiting, hematochezia, and the source of the bleeding was identified. The site of bleeding (upper or lower) and the causative disease were also investigated from endoscopic findings and/or imaging including computed tomography. The severity of GIB was defined as serious in case of BARC criteria type 3 and above. In addition, background factors included age at prescription, sex, weight, comorbidities (hypertension, diabetes, dyslipidemia, hyperuricemia, chronic kidney disease, chronic heart failure, ischemic heart disease, malignant disease, liver cirrhosis, chronic obstructive pulmonary disease), concomitant medications (nonsteroidal anti-inflammatory drugs (NSAIDs), antithrombotic agents, proton pump inhibitors), CHADS2 score, CHA2DS2-VASc score, HAS-BLED score, and status at the time of the event. All information was collected from medical records.

Endpoints were clinically relevant bleeding (including gastrointestinal bleeding), MACCE, and death. Details of gastrointestinal bleeding for each drug were also investigated. The observation period was measured in months: from the month of the first prescription to the month of the last prescription or event onset. Patients who had not been seen for more than 6 months without a clear reason (change of doctor or termination of treatment on the decision of the doctor) were considered as dropout cases.

Statistical analysis was performed using SPSS version 28 (IBM Japan, Tokyo, Japan). Kaplan–Meier curves were used to compare frequencies among multiple groups. A generalized linear model was used to adjust for background factors using the inverse weighting method of propensity scores calculated from background factors for each case, which was expressed as odds ratios and 95% confidence intervals.

## 3. Results

The median age of subjects was 73 years (interquartile range (IQR), 65–80 years), 622 (64%) were male, median height was 1.62 m (IQR, 1.55–1.68 m) and median weight was 62 kg (IQR, 52–70 kg). Comorbidities included hypertension in 808 patients (83%), diabetes in 253 (26%), dyslipidemia in 480 (49%), chronic kidney disease in 521 (53%), chronic heart failure in 560 (57%), ischemic heart disease in 245 (25%), cerebrovascular disease in 150 (15%), peripheral arterial disease in 60 (6%), and advanced malignant tumor in 85 (9%). Median CHADS2 score was 2 (IQR, 2–3), median CHA2DS2-VASc score was 4 (IQR, 3–5), and median HAS-BLED score was 3 (IQR, 3–4). Concomitant medications included low-dose aspirin in 200 (20%), ADPP2Y12 receptor antagonists in 128 (13%), NSAIDs in 30 (3%), and proton pump inhibitors in 535 (55%). Table 1 shows background factors by the DOAC prescribed.

The overall observation period was 2499 person-years. During the observation period, MACCE occurred in 102 patients (4.1/100 person-years) and clinically significant bleeding occurred in 107 patients (4.3/100 person-years) with gastrointestinal bleeding in 60 patients (2.4/100 person-years) and all-cause death in 37 patients (1.5/100 person-years). Most of the patients with GIB received an endoscopy. In particular, all patients with serious GIB underwent esophagogastroduodenoscopy or colonoscopy or both, and the medical team identified the causative lesion. Dropout was seen in 12 cases (0.5/100 person-years). Table 2 shows observations for each drug.

**Figure 2:**
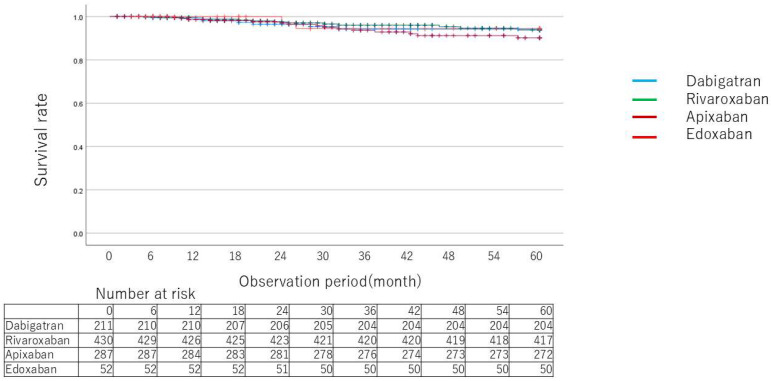
Kaplan–Meier curves for event probability of death by type of direct oral anticoagulants.

**Figure 3:**
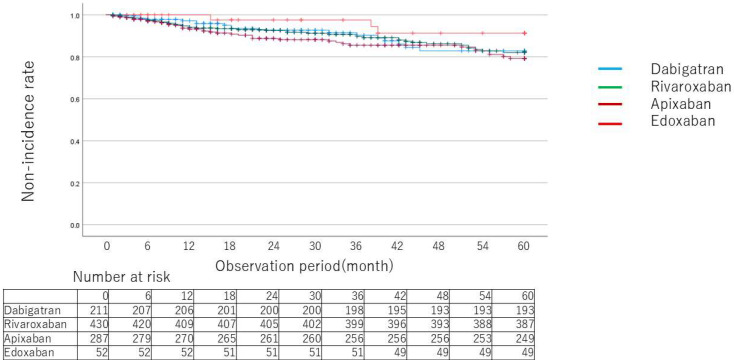
Kaplan–Meier curves for event probability of major cerebrovascular and cardiovascular events by type of direct oral anticoagulants.

**Figure 4:**
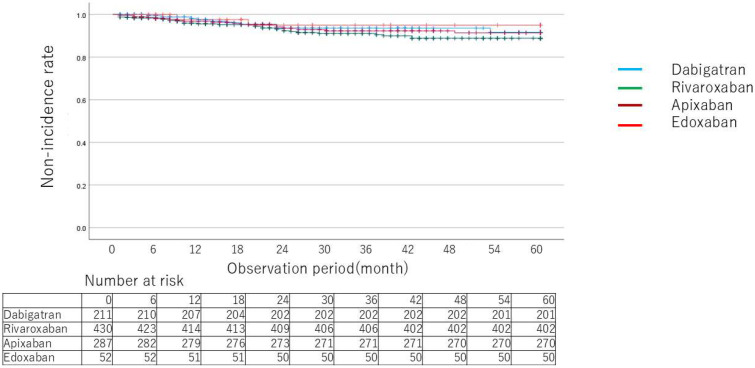
Kaplan–Meier curves for event probability of gastrointestinal bleeding by type of direct oral anticoagulants.

Kaplan–Meier curves for death (Figure 2), MACCE (Figure 3), and relevant gastrointestinal bleeding (Figure 4) showed no significant differences between drugs. Table 3 and Table 4 show the breakdown of causative diseases for gastrointestinal bleeding. Of the 60 cases of gastrointestinal bleeding, 18 were from the upper gastrointestinal tract and 42 were from the lower gastrointestinal tract, indicating that bleeding tended to more frequently arise from the lower part. Peptic ulcer accounted for half of the cases in the upper part, all of which were serious. Only one case was treated with concomitant proton pump inhibitors. Malignant disease was the second most common cause, followed by inflammation and anastomotic bleeding after surgical treatment, but severe cases were rare except for peptic ulcer. In the lower gastrointestinal tract, colonic diverticular hemorrhage was the most frequent, which was followed by telangiectasia and hemorrhage from hemorrhoids. Compared to peptic ulcer, bleeding from hemorrhoids tended to be less serious. A certain number of cases of bleeding after endoscopic treatment were also observed. As for the characteristics of each drug, there were no apparent differences in sex, age or HAS-BLED score between drugs. Lower gastrointestinal hemorrhage accounted for the majority of cases for all drugs with a marked trend for dabigatran. Lower gastrointestinal bleeding occurred in all but one case among patients taking dabigatran. For Xa inhibitors, colonic diverticular hemorrhage was the most common with severe cases accounting for about half of cases. Table 5 shows comparisons (hazard ratios) between drugs for relevant gastrointestinal bleeding. Rates of gastrointestinal bleeding were compared using a generalized linear model after adjusting for background factors using the inverse weighted method of propensity scores calculated from background factors.

## 4. Discussion

This study revealed five main findings. First, gastrointestinal bleeding accounted for more than half of all bleeding episodes among DOAC users. Second, lower gastrointestinal bleeding was more common than upper gastrointestinal bleeding, particularly among patients taking dabigatran. Third, peptic ulcer accounted for half of the episodes of upper gastrointestinal bleeding, all of which were severe. Fourth, colonic diverticulum, telangiectasia, and hemorrhoids were the most common cause of lower gastrointestinal bleeding, in that order. Fifth, no significant differences in the rate of gastrointestinal bleeding were seen by drug type.

This study was conducted by recruiting subjects from a list of prescribers. In Japan, DOACs are also administered to patients with deep vein thrombosis and pulmonary artery thrombosis, which are indications other than nonvalvular atrial fibrillation, but each condition has different risk factors, dosages and administration methods. The aim of this study was to evaluate the risk of adverse events in patients with nonvalvular atrial fibrillation, a common background condition, and to exclude patients receiving treatment for their underlying condition. The study was also designed to evaluate the occurrence of adverse events in patients on stable DOACs. Hospitalized patients were excluded from the study because they often have some acute problems that may influence the presence or absence of adverse events. Patients who had been taking the medication for less than one month were also excluded from the study because they had not been seen by a doctor since starting treatment, and their adherence to the medication had not been confirmed.

The majority of gastrointestinal bleeding events among DOAC users were consistent with what has been shown in previous reports. While the frequency and severity of bleeding at other sites tends to be lower with DOACs compared to warfarin, gastrointestinal bleeding is more common with DOACs in randomized controlled trials, and real-world data often report similar rates [10,11,12,13,14]. Our previous study reported that gastrointestinal bleeding was the most frequent type of bleeding, and a similar trend was seen in the present study [15]. The reason for this high incidence of gastrointestinal bleeding among DOAC users could be that conventional anticoagulants exert systemic effects after being absorbed from the gastrointestinal tract, whereas with DOACs, in addition to showing systemic effects, residual components not absorbed from the gastrointestinal tract have been suggested to exert effects locally and cause hemorrhage [19,20]. This suggests that the drug may remain in the gastrointestinal tract for longer. Ingason et al. reported less upper gastrointestinal bleeding in patients taking DOACs compared to those on the oral anticoagulant warfarin, but the same frequency for bleeding events involving the lower gastrointestinal tract, suggesting that pharmacokinetic differences may be responsible [35]. It is important to recognize that appropriate response to gastrointestinal bleeding in DOAC users poses a challenge that affects disease management.

For upper gastrointestinal bleeding, bleeding from peptic ulcers accounted for the majority of cases, all of which were severe. The overall rate of patients taking PPIs was about 50%, but the rate among patients with peptic ulcer bleeding was only 11%. An effect of PPIs in achieving peptic ulcer control with antithrombotic agents has also been reported [15,37,38,39]. Patients at high risk of upper gastrointestinal bleeding (i.e., those with a history of peptic ulcer or after peptic tract surgery) have been recommended to undergo evaluation for gastrointestinal risk before starting the administration of antithrombotic agents, and appropriate measures such as concomitant PPIs should be taken.

The main sources of lower gastrointestinal bleeding were colonic diverticulum, telangiectasia, and hemorrhoids. Previous reports have not provided detailed information on the causative agents of bleeding, and we believe such data are valuable. Regarding the lower gastrointestinal tract, patients taking DOACs are reportedly less likely to experience severe events than those taking warfarin [40,41]. The present study likewise encountered relatively few severe cases. However, it should be noted that about half of the cases of diverticular hemorrhage were severe, and the frequency of that event was high.

In the present study, no significant differences were evident in the rate or severity of gastrointestinal bleeding by DOAC type. Previous reports have suggested that differences may exist in gastrointestinal bleeding due to the significant differences in pharmacokinetic characteristics [19,20]. Dabigatran, a thrombin inhibitor, is less bioavailable than other DOACs, which are Xa inhibitors, and a large proportion of the drug remains in the gastrointestinal tract until being excreted without being absorbed. For rivaroxaban, an Xa inhibitor, a higher peak blood concentration is required with a higher once-daily dose to achieve efficacy, and that drug is reportedly more effective than twice-daily apixaban and edoxaban in reducing bleeding, particularly among patients with lower gastrointestinal bleeding. Concerns have been raised that this drug may be more prone to cause bleeding, particularly gastrointestinal bleeding, than apixaban and edoxaban, which are taken twice daily. Recently accumulated real-world data have supported such concerns [21,22,23,24,25,26,27,28,29,30,31]. However, scattered reports have described no differences between drugs, and no consensus has been reached [32,33,34]. In the present study, after adjusting for background factors as much as possible, we found no significant differences between drugs. However, the risk ratio was higher for rivaroxaban than for any of the other drugs. This may have been due to the small number of subjects included in this study, and future studies with larger numbers of patients are warranted.

Based on the present results, we believe that we can present the following strategy in order to control gastrointestinal bleeding during DOAC administration. First, in the upper gastrointestinal tract where preventive measures are clear, it is necessary to conduct a preliminary evaluation with an upper gastrointestinal endoscopy as much as possible, bearing in mind that bleeding will become more severe, and PPI should be used in combination with people with a history of ulceration. Next, keeping in mind that the lower gastrointestinal tract is more frequent but not more severe, the progression of anemia and the presence or absence of overt bleeding should be fully evaluated, and if there is a concern about bleeding, endoscopic evaluation and risk factors (concomitant medications, etc.) should be attempted. In this way, we think that it is possible to reduce gastrointestinal bleeding during DOAC treatment and provide safer and higher-quality treatment by separating the upper and the lower gastrointestinal tract.

Limitations of this study include the retrospective, single-center study design and the small number of subjects. In particular, the number of subjects was smaller for edoxaban than for other drugs, and it may not have been sufficient to allow sufficient evaluation of the event. In the future, it will be necessary to collect a wider range of information through prospective studies and compare it with existing scores such as the Rockall score or Glasgow–Blatchford score to improve the predictability of gastrointestinal bleeding, as this is a backward-looking study, and more detailed information, such as vital signs during bleeding, is not available. We believe that it is necessary to obtain a higher level of information. In addition, as the information was obtained from a single center, selection bias may have influenced the results in terms of patient and drug selection, and a multicenter study is desirable in the future to make the information obtained in this study more universal.

## 5. Conclusions

Here, we clarified that gastrointestinal bleeding was frequent among patients with NVAF taking DOACs, especially from the lower alimentary tract. The tendency was more pronounced with dabigatran. In the upper gastrointestinal tract, severe peptic ulcer bleeding is common and requires caution. In the lower gastrointestinal tract, colonic diverticulum, telangiectasia, and hemorrhoids were the most common cause of bleeding. We believe that this study will give precious information about GIB during DOAC therapy for clinicians who take care of such patients in the clinical setting.

## Figures and Tables

**Table 1:** Background characteristics of the subjects by drugs.

Drug	Dabigatran	Rivaroxaban	Apixaban	Edoxaban
Number of patients	211	430	287	50
Sex (male/female)	149/62	270/160	173/114	30/20
Age (year); median (IQR)	71 (64–77)	72 (66–79)	78 (69–83)	69 (62–78)
Height (m); median (IQR)	1.65 (1.57–1.70)	1.62 (1.55–1.69)	1.61 (1.52–1.68)	1.65 (1.53–1.69)
Weight (kg); median (IQR)	64 (54–71)	62 (53–73)	60 (51–69)	64 (52–70)
Comorbidity				
Hypertension; *n* (%)	182 (86)	355 (83)	230 (80)	41 (82)
Dyslipidemia; *n* (%)	88 (42)	217 (50)	149 (52)	26 (52)
Diabetes Mellitus; *n* (%)	46 (22)	120 (28)	78 (27)	9 (18)
Chronic heart disease; *n* (%)	145 (69)	230 (53)	161 (56)	24 (48)
Ischemic heart failure; *n* (%)	38 (18)	104 (24)	91 (32)	12 (24)
Peripheral arterial disease; *n* (%)	7 (3)	28 (7)	22 (8)	3 (6)
Cerebrovascular disease; *n* (%)	33 (16)	69 (16)	45 (16)	3 (6)
Chronic kidney disease; *n* (%)	92 (44)	209 (49)	192 (67)	28 (56)
Malignancy	13 (6)	45 (10)	24 (8)	3 (6)
History of gastrointestinal bleeding; *n* (%)	3 (1)	6 (1)	5 (2)	0 (0)
CHADS2 score; median (IQR)	2 (2–3)	2 (2–3)	2 (2–3)	2 (2–3)
CHADS2-VASc score; median (IQR)	4 (3–5)	4 (3–5)	4 (3–5)	4 (3–5)
HAD-BLED score; median (IQR)	3 (3–4)	3 (3–4)	3 (3–4)	3 (3–4)
Concomitant medicine				
Low-dose aspirin; *n* (%)	46 (22)	86 (20)	60 (21)	8 (15)
P2Y12 RA; *n* (%)	12 (6)	61 (14)	49 (17)	6 (12)
NSAIDs; *n* (%)	7 (3)	12 (3)	9 (3)	2 (4)
PPI; *n* (%)	95 (45)	233 (54)	177 (61)	30 (58)

Abbreviations: IQR, interquantile range; *n*, number of patients; P2Y12 RA, adenosine 2 phosphate receptor P2Y12 receptor antagonist; PPI, proton pump inhibitor.

**Table 2:** Observational data by drugs.

Drug	Dabigatran	Rivaroxaban	Apixaban	Edoxaban
Observation period; patient-year	488.5	1067.8	774.6	168.3
Clinically relevant bleeding (/100 patient·year)	20 (4.1)	54 (5.1)	30 (3.9)	3 (1.8)
Gastrointestinal bleeding (/100 patient·year)	10 (2.1)	31 (2.9)	17 (2.2)	2 (1.2)
MACCE (/100 patient·year)	18 (3.7)	43 (4.0)	38 (4.9)	3 (1.8)
Death (/100 patient·year)	7 (1.4)	13 (1.2)	15 (1.9)	2 (1.2)
Drop out (/100 patient·year)	4 (0.8)	5 (0.5)	3 (0.4)	0 (0)

Abbreviations: MACCE, major cerebrovascular and cardiovascular events.

**Table 3:** Characteristics of gastrointestinal bleeding by drugs.

	Dabigatran	Rivaroxaban	Apixaban	Edoxaban
Number of patients	211	430	287	50
Number of patients with GIB (%)	10 (4.7)	31 (7.2)	17 (5.9)	2 (4.0)
Upper GIB	1	12	5	0
Lower GIB	9	19	12	2
Male/Female	5/5	17/14	11/6	2/0
Age; median(range)	78 (66–92)	75 (57–96)	76 (49–93)	68 (61–74)
HAS-BLED score; median (range)	3 (2–5)	3 (1–5)	3 (1–5)	2 (1–3)

Abbreviations: GIB; gastrointestinal bleeding.

**Table 4:** Cause of serious gastrointestinal bleeding.

	Dabigatran	Rivaroxaban	Apixaban	Edoxaban
Number of patients with serious GIB (serious cases/all cases, %)	3 (30%)	12 (39%)	7 (41%)	1 (50%)
Cause of serious bleeding; number of patients	Peptic ulcer; 1, Colonic telangiectasia; 1, Colitis; 1	Peptic ulcer; 7, Colonic diverticula; 2, Colonic telangi- ectasia; 1, Hemorrhoids; 1, Postpolypectomy for colonic polyp; 1	Peptic ulcer; 1, Gastric cancer; 1, Colonic diverticula; 3, Colon cancer; 1, Postpolypetomy for colonic polyp; 1	Colonic diverticula; 1

Abbreviations: GIB; gastrointestinal bleeding.

**Table 5:** Comparison of rate of gastrointestinal bleeding with drugs.

Drug	Number of Patients	Bleeding Rate (/100 Patient-Year	Versus Dabigatran HR (95%CI), *p*-Value	Versus Rivaroxaban HR (95%CI), *p*-Value	Versus Apixaban HR (95%CI), *p*-Value	Versus Edoxaban HR (95%CI), *p*-Value
Dabigatran	211	2.05	N/A	0.69 (0.32–1.48), *p* = 0.34	0.86 (0.37–2.03), *p* = 0.74	1.14 (0.22–5.86), *p* = 0.88
Rivaroxaban	430	2.90	1.45 (0.67–3.11), *p* = 0.34	N/A	1.30 (0.68–2.46), *p* = 0.43	1.91 (0.44–8.34), *p* = 0.39
Apixaban	287	2.19	1.16 (0.49–2.72), *p* = 0.74	0.77 (0.41–1.47), *p* = 0.43	N/A	1.78 (0.40–8.10), *p* = 0.45
Edoxaban	50	1.19	0.88 (0.17–4.52), *p* = 0.88	0.52 (0.12–2.28), *p* = 0.39	0.56 (0.12–2.53), *p* = 0.45	N/A

Figures are adjusted hazard ratios (95% confidence interval) and *p*-values evaluated by a generalized linear estimating equation model using the propensity score (age, sex, weight, hypertension, diabetes mellitus, dyslipidemia, chronic heart failure, ischemic heart disease, cerebrovascular disease, peripheral arterial disease, chronic obstructive pulmonary disease, liver cirrhosis, advanced malignancy, chronic kidney disease) weighting by the inverse probability weighting method. Abbreviations: HR; hazard ratio, CI; confidence interval, N/A; not available.

## Data Availability

The data supporting the findings of this study are available on request from the corresponding author, Takatsugu Yamamoto (ymmt@med.teikyo-u.ac.jp) at Teikyo University School of Medicine. The data are not publicly available due to the inclusion of information that could compromised the privacy of research participants.
